# Locally advanced carcinoma of the cecum presenting as a right inguinal hernia: a case report and review of the literature

**DOI:** 10.1186/1752-1947-7-206

**Published:** 2013-08-14

**Authors:** Roberto Luca Meniconi, Giovanni Vennarecci, Pasquale Lepiane, Andrea Laurenzi, Roberto Santoro, Marco Colasanti, Mario Antonini, Giuseppe Maria Ettorre

**Affiliations:** 1 Division of General Surgery and Organ Transplantation, S. Camillo Hospital, POIT S. Camillo-INMI Lazzaro Spallanzani, Circonvallazione Gianicolense 87, Rome, Italy; 2 Intensive Care Unit, INMI Lazzaro Spallanzani, POIT S. Camillo-INMI Lazzaro Spallanzani, Rome, Italy

**Keywords:** Cecum carcinoma, Inguinal hernia, Intrasaccular tumors, Right colectomy

## Abstract

**Introduction:**

An inguinal hernia is a common surgical disease in elderly patients, but an association with intra-abdominal malignancies is rare.

**Case presentation:**

We report a case of a 78-year-old Caucasian woman presenting with a right inguinal mass suspected to be an irreducible hernia. A computed tomography scan showed the presence of the cecum in her inguinal canal, with an irregular thickening of the cecal wall suggesting a neoplasm within the inguinal hernia. A colonoscopy was not completed owing to the huge involvement of the cecum into the hernia sac. A laparotomy was performed, at which time the cecum was herniated through her right inguinal canal and the cecal tumor had infiltrated her abdominal wall and femoral artery. A right inguinal incision was necessary for good vascular control and to carry out an *en bloc* resection of the tumor with the inguinal wall. A right colectomy was performed and the inguinal wall repaired. The postoperative course was uneventful and our patient received adjuvant radiochemotherapy.

**Conclusion:**

We describe a rare case of a locally advanced cecal tumor presenting as a right inguinal hernia. Both diagnosis and surgical treatment in elderly patients represent a challenge for the surgeon in cases of aggressive tumors as reported in this paper.

## Introduction

Intra-abdominal malignancies presenting as inguinal hernias are rare and have been classified as saccular or intrasaccular tumors based on their relationship with the inguinal sac [1]. Saccular tumors are tumors of the peritoneal surface of the sac that can be primary (such as mesothelioma) or secondary (for example, peritoneal carcinomatosis). Intrasaccular tumors are primary tumors of abdominal organs (for example, colonic cancer) contained within the inguinal sac. Intrasaccular tumors are rarer and the most commonly reported site is the left inguinal hernia containing a sigmoid cancer [2,3]. The treatment of this rare condition is not standardized and the correct surgical strategy may represent a challenge for the surgeon, especially in cases of advanced tumors. We report a rare case of an intrasaccular tumor due to an aggressive cecal cancer presenting within a right inguinal hernia in an elderly woman.

## Case presentation

**Figure 1:**
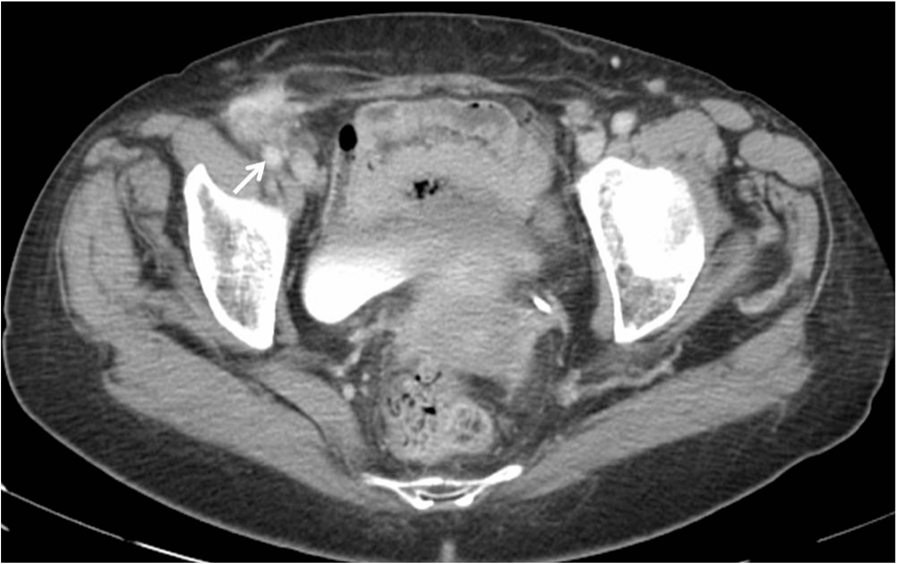
Computed tomography scan shows the cecal wall thickness within a right inguinal hernia involving the femoral artery (arrow).

A 78-year-old Caucasian woman was admitted to our hospital presenting with a painful mass in her right groin, without fever, rectal bleeding or any other bowel symptoms. She had no history of malignant diseases. A physical examination showed an irreducible palpable mass in her right inguinal region protruding through the external inguinal ring. Routine blood examinations showed she had chronic anemic status with an hemoglobin value of 8.9g/dL. Tumor markers values of carcinoembryonic antigen and carbohydrate antigen 19.9 were 4ng/mL and 24U/mL, respectively. A computed tomography scan of her abdomen showed the presence of the cecum in her inguinal canal, with an irregular thickening of its wall suggesting a cecal neoplasm within the inguinal hernia involving the femoral vessels (Figure 1).

**Figure 2:**
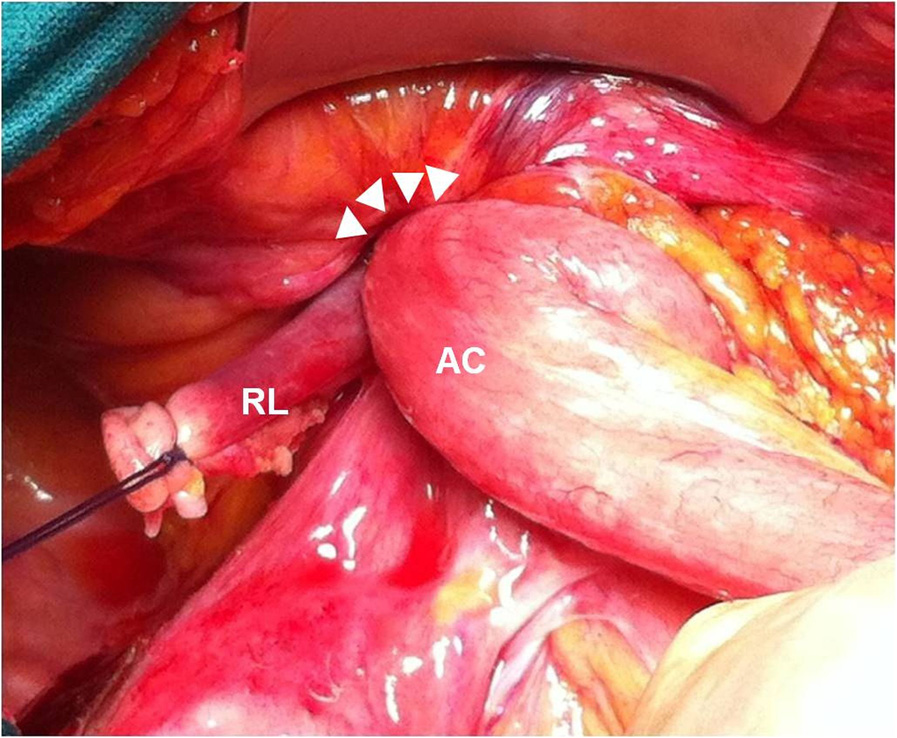
**The cecal tumor is herniated into the right inguinal canal (arrowheads), infiltrating the abdominal wall and the round ligament.** AC, ascending colon; RL, round ligament.

A colonoscopy was not completed due to the large involvement of the cecum into the hernia sac. Surgical exploration was made through a midline laparotomy that confirmed the herniation of the cecum into her right inguinal canal (Figure 2), without the possibility of reduction in her abdominal cavity owing to the tumor infiltration into her abdominal wall and inguinal structures. Thus, a right inguinal incision was made, which revealed an aggressive cecal tumor infiltrating her inguinal wall, right round ligament and femoral artery. The mass was resected *en bloc*, with complete vascular control of her femoral vessels, and reduced into her abdomen.

**Figure 3:**
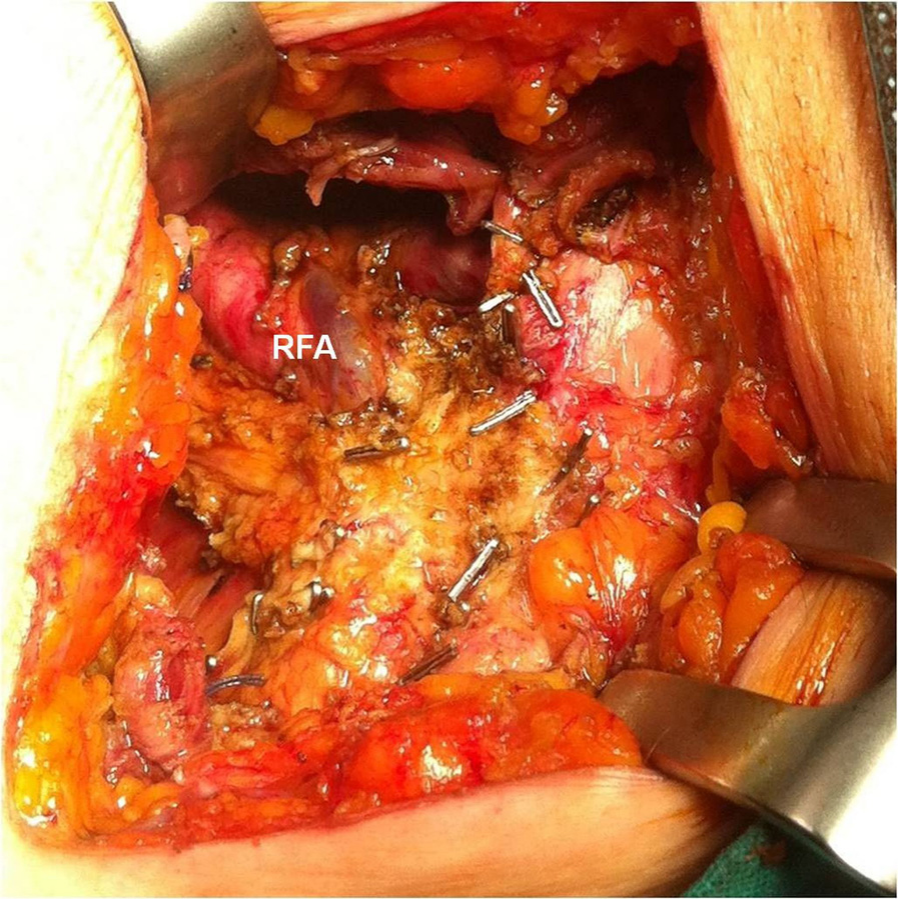
**After resection, the operative field of the femoral region is marked by surgical clips for further radiation therapy.** RFA, right femoral artery.

**Figure 4:**
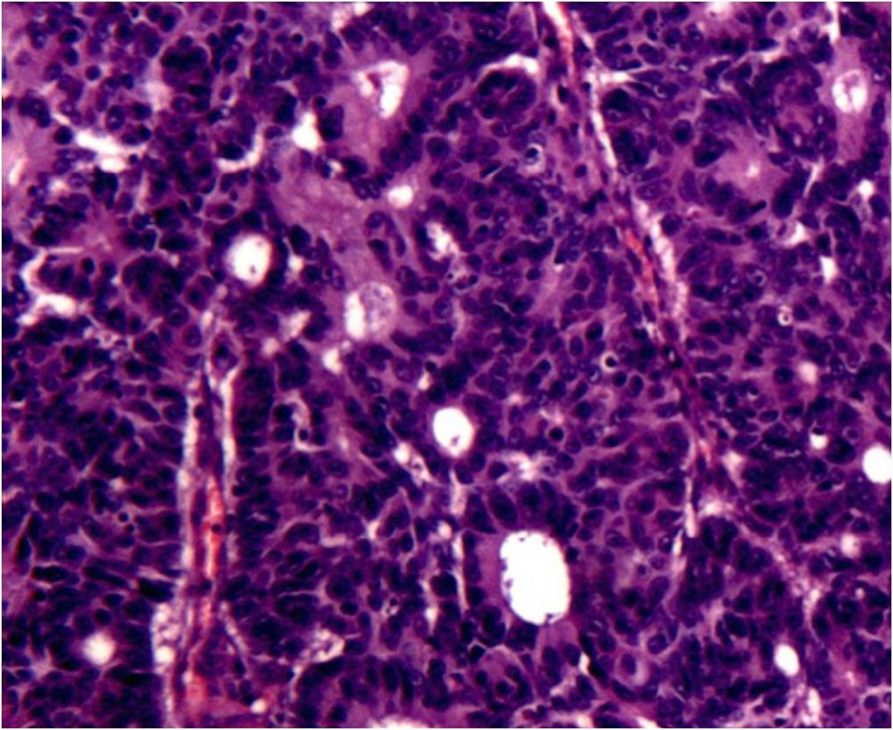
**Microscopy image showing pleomorphism and hyperchromatism of tumor cells with nuclear atypia and low grade gland formation.** Note the presence of hemorrhage and hemosiderin. (Hematoxylin-eosin staining, ×50).

Finally, a right colectomy was performed with a manual ileocolic anastomosis and her inguinal wall was repaired by a direct suture without mesh. The operative field of the femoral region was also marked with surgical clips for further postoperative radiation therapy (Figure 3). Histopathological examination of the specimen showed a poorly differentiated adenocarcinoma of the large bowel with lymphovascular and perineural invasion, microscopic involvement of the resection margins, and metastases of 8 out of 17 regional lymph nodes (pT4b pN2b M0, R1; Stage IIIC; Dukes C3) (Figure 4). The postoperative course was uneventful and our patient received adjuvant radiochemotherapy. Six months after surgery, our patient is alive and disease free.

## Discussion

Inguinal hernias and colonic malignancies are frequent diseases in the elderly population, but their association is relatively rare. Two previous literature reviews [3,4] revealed that the sigmoid colon was involved in most cases and all patients were male. Out of 28 patients reported, only four had a cecal tumor, presenting in all cases as a right long-standing inguinal hernia that become painful or incarcerated. In our case, a female patient recently noticed a mass in her right groin, without any symptoms or signs of obstruction; she had no history of inguinal hernia or primary malignancy, only a general asthenia. A correct diagnosis in these cases may be difficult, especially in elderly patients, and computed tomography should always be performed to confirm the suspicion of an underlying malignancy. A colonoscopy may present with negative results as in our case owing to the involvement of the colon into the hernia.

The best surgical treatment is not clear and depends on the patient’s characteristics (age, general condition), local findings (infiltration of organs or vessels) and the surgeon’s experience [4]. In the majority of the reported cases, a laparotomic resection of the colon followed a traditional inguinal repair through two separate incisions [2,4]. In cases of perforation or occlusion, most authors performed a colonic resection through the inguinal incision to prevent the peritoneal cavity from contamination and completed the operation via a midline laparotomy [3,5]. Other authors described a transverse left iliac fossa incision for a sigmoid cancer incarcerated into a left inguinal hernia [6]. More recently, a laparoscopic approach has been described in one case [7]: the tumor was reduced and resected by laparoscopy, while the inguinal defect was repaired by a traditional approach. Despite our experience with laparoscopic colorectal surgery, and considering the advanced local status of the tumor, we decided to perform a midline laparotomy and found an irreducible cecal tumor within the inguinal canal. A secondary inguinal incision was necessary to take control of vascular structures, performing an *en bloc* resection of the tumor with the inguinal wall. Marking the operative field with metallic clips for postoperative radiotherapy could be a good solution in cases of aggressive tumors suitable for adjuvant radiotherapy after microscopically incomplete resections. As to neoadjuvant treatment, we decided to address the patient directly to surgery due to the risk of obstruction and tumor progression during preoperative radiochemotherapy, but it might otherwise be considered, especially when positive margins are expected after resection.

## Conclusions

Inguinal hernias containing a colonic malignancy are not frequent, but should be evaluated in elderly patients presenting with an irreducible mass in the inguinal region associated with gastrointestinal symptoms or non-specific features such as asthenia or anemia. The surgical treatment can be achieved either by an open or laparoscopic approach but should always respect the oncological standards of a radical resection, especially when adjacent structures are involved.

## Consent

Written informed consent was obtained from the patient for publication of this case report and any accompanying images. A copy of the written consent is available for review by the Editor-in-Chief of this journal.

## Competing interests

The authors declare that they have no competing interests.

## Authors’ contributions

RLM collected clinical data, reviewed the literature and drafted the manuscript; GV reviewed the manuscript for intellectual content; PL, AL, RS, MC and MA were involved in the care of the patient; GME supervised the whole process. All authors read and approved the final manuscript.
